# The Seventh Annual Commencement of the University of Maryland Dental Department

**Published:** 1889-03

**Authors:** 


### Editorial, Etc.

---

THE SEVENTH ANNUAL COMMENCEMENT OF THE UNIVERSITY OF MARYLAND DENTAL DEPARTMENT.—The commencement exercises of the University of Maryland Dental Department took place at the Lyceum Theatre, Baltimore, on Wednesday evening, March 13th, 1889. The auditorium of this beautiful building was filled with ladies and gentlemen to witness the exercises, and the flowers presented to the graduates by their friends were handsomely arranged in many designs and very numerous.

The beautiful stage, designed by Dr. A. J. Volck, a prominent member of the dental profession of Baltimore, who was present on this occasion, was filled with the Faculty, Graduates and invited guests; among the latter being prominent citizens and professional gentlemen from New York, Pennsylvania, Virginia, South Carolina, West Virginia and Maryland. The exercises began with prayer by the Rev. Charles C. Cooke. The mandamus with the names of the graduates was read by the Dean, Prof. Ferdinand J. S. Gorgas, M. D., D. D. S. The degree of "Doctor of Dental Surgery" was then conferred upon the following members of the graduating class, by authority from the Board of Regents, Hon. Severn Teackle Wallis, L. L. D., Provost.

| | |
|---|---|
| Charles G. Aven, | Virginia |
| Augustine Pennington Badger, | Maryland |
| Eugene James Bailey, | South Carolina |
| Victor Durand Barbot, | South Carolina |
| Joseph Percy Blair, | Virginia |
| Frank Beck | Pennsylvania |
| Kelly Ragland Bragg, | Missouri |
| H. Wood Campbell, | Virginia |
| Thomas S. D. Covington, | Virginia |
| August Adolph Theodor Wilhelm Cuny, | Germany |

| | |
|---|---|
| E. Douglas Davis, - - - - | W.Virginia |
| Henry Davis, M. D., - - - - | Missouri |
| Edwin R. Dodson, M. D., - - | Maryland |
| Pearl Louis Ellis; - - - - | Vermont |
| William Lafayette Fish, - - - | New Jersey |
| Henry Augustus Free, - - | Pennsylvania |
| David Goebricher, - - - - | Maryland |
| Joseph A. Haas, - - - - | New York |
| E. Patterson Hayes, - - - | Pennsylvania |
| Reuben Benjamin Hills, - - | Massachusetts |
| William Henry Holland, - - | South Carolina |
| John Whiteford McKinnon, - - | Pennsylvania |
| William Lee Miller, - - - - | W. Virginia |
| J. England Molony, - - - | South Carolina |
| Solomon L. Nigolosian, - - - | Asia Minor |
| Frank Merriman Oldham, - - | South Carolina |
| Czeslaus Opielinski, - - - | Germany |
| Geo. B. Patterson, - - - | North Carolina |
| Frank Zea Pirkey, - - - - | California |
| Benson S. Roberts, - - - - | Bermuda |
| Fritz Franz Wilhelm Schloendorn, - - | Germany |
| Joseph B. Sharp, - - - - | New Jersey |
| Archie Carver Shoemaker, - - | Pennsylvania |
| Benjamin Simons, - - - - | South Carolina |
| Hampton K. Smith, - - - | South Carolina |
| Cornelius van der Hoeven, M. D., - - | Holland |
| William J. Warnock, - - - | South Carolina |
| Murray V. Wright, M. D., - | New Hampshire |

After the conferring of the degrees, the awards of the prizes were announced by the Dean, and the prizes, fourteen in number, were presented by Prof. J. Edwin Michael, M. D., the dean of the University School of Medicine, and professor of anatomy in the Dental Department, as follows:

University Gold Medal, to Victor D. Barbot, of South Carolina, for the highest number of votes at the final examination; honorable mention, Hampton K. Smith, of South Carolina.

S. S. White prize of dental engine, to E. Patterson Hayes, of Pennsylvania, for the best two fillings of cohesive and noncohesive gold; honorable mention, David Goebricher, of Maryland.

Francis Arnold prize of set of forceps, to Fritz F. W. Schloendorn, of Germany, for best full set of gum teeth on metal.

Snowden & Cowman prize of set of Chapin A. Harris' forceps, to Cornelius van der Hoeven, M. D., of Holland, for best full set of continuous gum work; honorable mention, Joseph A. Haas, of New York.

Dr. James H. Harris gold medal, to Victor D. Barbot, of South Carolina, for the best filling of non-cohesive gold.

Dr. F. J. S. Gorgas gold medal, to William Lee Miller, of West Virginia for the best filling of cohesive gold; honorable mention, E. Douglas Davis, of West Virginia.

Dr. Jno. C. Uhler gold medal, to August A. T. W. Cuny, of Germany, for the best partial set on metal.

Dr. Charles L. Steel gold medal, to August A. T. W. Cuny, of Germany, for the best bridge and crown work.

Dr. I. H. Davis gold medal, to David Goebricher, of Maryland for the best combination set of vulcanite and metal.

S. S. White prize set of instruments, to August A. T. W. Cuny, of Germany, for best vulcanite set.

Dental Department gold medal, to Fritz F. W. Schloendorn, of Germany, for the best set on celluloid base.

Dr. Frank L. Wood gold medal, to George B. Patterson, of North Carolina, for the best specimen tooth gold filling; honorable mention, Fritz F. W. Schloendorn, of Germany.

Class gold medal, to August A. T. W. Cuny, of Germany, for the best specimen set of teeth; honorable mention, Fritz F. W. Schloendorn, of Germany.

Dr. D. Genese prize, to Fritz F. W. Schloendorn, of Germany, for an artistic plaster model of the cranium and jaws and bridge-work connection.

The address to the graduates was delivered by Dr. J. B. Patrick, of Charleston, S. C., one of the most prominent dental practitioners in the South, whose manner of delivery was that of a practiced orator, and his address a most acceptable and interesting comparison of the dentistry of the past with that of the present, and an earnest appeal to the graduates to practice their profession in a manner worthy of the old and renowned institution, by whose authority they enter upon the practice of dental science. The class oration was delivered by Dr. Hampton K. Smith, of S. C., a member of the graduating class, and its delivery was such as to prove to his audience that his classmates had been very fortunate in the selection of their orator.

The number of matriculates of the session 1888-89, was one hundred and twenty.

Following the commencement exercises, the annual meeting of the Alumni Association was held, at which Dr. E. P. Beadles, of Danville, Va., was elected President, and Dr. Chas. F. Dinger, of Baltimore, Secretary. The annual banquet of the Alumni Association was held at the Howard House, the toasts being responded to by Profs. Gorgas, Michael, Atkinson, Coale, Uhler and Harlan, of the Dental Department Faculty, and Dr. Beadles, President of the Alumni Association, Dr. H. K. Smith, of the graduating class, Dr. Richard Grady, of the Maryland State Board of Dental Examiners, and by Drs. D. Genese, W. A. Montell and others. The menu was elaborate, and well served, and the occasion will long be remembered by all who participated.

